# PREVENT Equation: The Black Sheep among Cardiovascular Risk Scores? A Comparative Agreement Analysis of Nine Prediction Models in High-Risk Lithuanian Women

**DOI:** 10.3390/medicina60091511

**Published:** 2024-09-16

**Authors:** Petras Navickas, Laura Lukavičiūtė, Sigita Glaveckaitė, Arvydas Baranauskas, Agnė Šatrauskienė, Jolita Badarienė, Aleksandras Laucevičius

**Affiliations:** 1 Faculty of Medicine, Institute of Clinical Medicine, Vilnius University, 03101 Vilnius, Lithuania; lukaviciute.laura@gmail.com (L.L.); sigita.glaveckaite@santa.lt (S.G.); arvydas.baranauskas@santa.lt (A.B.); agne.satrauskiene@gmail.com (A.Š.); jolita.badariene@santa.lt (J.B.); 2 State Research Institute Centre for Innovative Medicine, 08410 Vilnius, Lithuania; aleksandras.laucevicius@santa.lt

**Keywords:** cardiovascular diseases, risk prediction models, women’s health, inter-model agreement

## Abstract

*Background and Objectives:* In the context of female cardiovascular risk categorization, we aimed to assess the inter-model agreement between nine risk prediction models (RPM): the novel Predicting Risk of cardiovascular disease EVENTs (PREVENT) equation, assessing cardiovascular risk using SIGN, the Australian CVD risk score, the Framingham Risk Score for Hard Coronary Heart Disease (FRS-hCHD), the Multi-Ethnic Study of Atherosclerosis risk score, the Pooled Cohort Equation (PCE), the QRISK3 cardiovascular risk calculator, the Reynolds Risk Score, and Systematic Coronary Risk Evaluation-2 (SCORE2). *Materials and Methods:* A cross-sectional study was conducted on 6527 40–65-year-old women with diagnosed metabolic syndrome from a single tertiary university hospital in Lithuania. Cardiovascular risk was calculated using the nine RPMs, and the results were categorized into high-, intermediate-, and low-risk groups. Inter-model agreement was quantified using Cohen’s Kappa coefficients. *Results:* The study uncovered a significant diversity in risk categorization, with agreement on risk category by all models in only 1.98% of cases. The SCORE2 model primarily classified subjects as high-risk (68.15%), whereas the FRS-hCHD designated the majority as low-risk (94.42%). The range of Cohen’s Kappa coefficients (−0.09–0.64) reflects the spectrum of agreement between models. Notably, the PREVENT model demonstrated significant agreement with QRISK3 (κ = 0.55) and PCE (κ = 0.52) but was completely at odds with the SCORE2 (κ = −0.09). *Conclusions:* Cardiovascular RPM selection plays a pivotal role in influencing clinical decisions and managing patient care. The PREVENT model revealed balanced results, steering clear of the extremes seen in both SCORE2 and FRS-hCHD. The highest concordance was observed between the PREVENT model and both PCE and QRISK3 RPMs. Conversely, the SCORE2 model demonstrated consistently low or negative agreement with other models, highlighting its unique approach to risk categorization. These findings accentuate the need for additional research to assess the predictive accuracy of these models specifically among the Lithuanian female population.

## 1. Introduction

Cardiovascular diseases (CVDs) represent a paramount health challenge globally, with a particularly pronounced impact on women. As the leading cause of morbidity and mortality among women worldwide, CVD accounts for up to 36% of all deaths in this demographic, overshadowing fatalities from all cancers combined [1,2]. This significant health concern persists despite the overall decline in age-standardized cardiovascular mortality rates over recent decades [3]. Notably, the incidence of coronary heart disease (CHD) in younger women exhibits a worrying uptrend [4,5], underpinning the intricate nature of CVD in women and its ties to factors such as ovarian function and hypertension [6].

The challenge of addressing CVD in women is compounded by issues of under-diagnosis, undertreatment, and under-representation in cardiovascular research [7]. These obstacles underscore the need for tailored risk assessment and prevention strategies specifically designed for women to mitigate the prevalence of CVD [8]. Despite the grim statistics—highlighting CVD as responsible for 8.94 million deaths annually and affecting 275 million women globally [9]—advancements in risk prediction models offer a glimmer of hope.

Risk prediction tools, integral for estimating the potential for fatal and non-fatal cardiovascular events, have evolved considerably. These tools enable healthcare professionals to formulate preemptive strategies and modify risk factors effectively [10,11]. The introduction of innovative prediction models like the Predicting Risk of Cardiovascular Disease EVENTs (PREVENT) equation, which considers novel risk factors, marks a significant stride in personalized healthcare [12]. Further, the integration of machine learning and deep learning techniques in cardiovascular risk prediction signifies a leap toward more accurate and individualized risk assessments [13].

Despite these advancements, the literature reveals a paucity of studies focusing on the agreement between risk categories among women, particularly in comparative analyses including novel models like PREVENT. This gap in research motivates the current study, which aims to conduct a comprehensive comparison of nine widely used cardiovascular risk prediction models. These include the PREVENT, the Systematic Coronary Risk Evaluation 2 (SCORE2), assessing cardiovascular risk using SIGN (ASSIGN), the Framingham Risk Score for Hard Coronary Heart Disease (FRS-hCHD), the Australian CVD risk score (AusCVDRisk), the Reynolds Risk Score (RRS), the Pooled Cohort Equation (PCE), the Multi-Ethnic Study of Atherosclerosis risk score (MESA), and the QRISK3 cardiovascular risk calculator (QRISK3). This study endeavors to illuminate the comparative agreement of these models in the context of female cardiovascular risk categorization.

## 2. Materials and Methods

### 2.1. Study Population/Criteria for Inclusion and Exclusion

The dataset encompassed individuals enrolled in the LitHiR (Lithuanian High Cardiovascular Risk primary prevention program), a government-sponsored initiative launched in Lithuania in 2006, aimed at the multifactorial reduction in cardiovascular (CV) risk among middle-aged individuals to avert the early onset of atherosclerosis [14]. The program targeted a population aged 40 to 65 years, without evident CVD, but diagnosed with metabolic syndrome (MetS), and these subjects were assessed from 2006 to 2023 at the tertiary care facility—Vilnius University Hospital Santaros Klinikos, located in Vilnius, Lithuania. Metabolic Syndrome was defined according to the updated criteria of the National Cholesterol Education Program Adult Treatment Panel III (NCEP ATPIII), which necessitated the presence of three or more of the following criteria: systolic blood pressure (SBP) of ≥130 mmHg or diastolic blood pressure (DBP) of ≥85 mmHg, or an existing hypertension diagnosis; a waist circumference of ≥88 cm for females and ≥102 cm for males; high-density lipoprotein (HDL) cholesterol levels of <1.29 mmol/L in females and <1.03 mmol/L in males; triglyceride (TG) levels of ≥1.7 mmol/L or special treatment is administered to reduce TG concentration; and diagnosed type 2 diabetes mellitus or fasting plasma glucose levels of ≥5.6 mmol/L [15].

Data for the present study were derived from the LitHiR program’s prospectively and uniformly collected dataset, which constituted the foundation for extracting the vital variables necessary for analysis. Inclusion criteria for subjects entailed having documented measures for key parameters, including low-density lipoprotein (LDL), high-density lipoprotein (HDL), total cholesterol (TC), systolic blood pressure (SBP), diastolic blood pressure (DBP), fasting glucose levels, creatinine, C-reactive protein (CRP), and the urine albumin–creatinine ratio, along with detailed family and medication history. Any subject lacking records for these crucial indicators was omitted from the analysis, a measure taken to maintain the integrity and accuracy of the risk assessment process.

For this investigation, the exclusion criteria encompassed individuals with a history of silent myocardial ischemia, coronary artery disease, transient ischemic attacks, peripheral artery disease, both ischemic and hemorrhagic strokes, oncological conditions, chronic or persistent arrhythmias, severe renal or liver dysfunction, significant psychiatric conditions, gout, as well as those who were pregnant, were undergoing therapy with xanthine oxidase inhibitors, or had drug addiction issues. This approach was adopted to eliminate confounding factors and ensure the study population was representative of individuals primarily at risk for metabolic syndrome without the influence of these complex comorbid conditions.

### 2.2. Risk Prediction Models

#### 2.2.1. Predicting Risk of Cardiovascular Disease EVENTs (PREVENT)

The PREVENT equation provides 10-year risk estimates for individuals of 30–79 years of age and provides 30-year risk estimates for individuals of 30–59 years of age. The algorithms were developed by the American Heart Association Cardiovascular-Kidney-Metabolic Scientific Advisory Group. The risk equation was derived and validated in a large, diverse sample of over 6 million individuals [12,16]. The calculation of the risk score was performed using a specific calculator available at: https://professional.heart.org/en/guidelines-and-statements/prevent-calculator (accessed on 1 March 2024). The 10-year risk of CVD was calculated. During the computation procedure, the inclusion of the Zip Code was not applicable.

#### 2.2.2. Systematic Coronary Risk Evaluation 2 (SCORE2)

SCORE2 is a risk assessment tool created to estimate the 10-year risk of both fatal and non-fatal CVD in individuals aged 40 to 69 years in Europe who have no prior history of CVD. This score represents a refinement of the original SCORE model, updated to include more recent data [17]. The SCORE2 model factors in various risk determinants such as age, gender, systolic blood pressure, non-HDL cholesterol levels, and smoking status. The calculation of the risk score was performed using a specific calculator available at: https://u-prevent.com/calculators/score2 (accessed on 2 June 2023). For this analysis, we employed a recalibrated version of the risk score tailored for use in areas identified as ‘very high risk’ by pertinent guidelines, without any alterations to the original computation process.

#### 2.2.3. Pooled Cohort Equations (PCE) Cardiovascular Risk Score

PCE was designed to estimate an individual’s likelihood of experiencing atherosclerotic CVD (ASCVD) over the course of a decade, utilizing data gathered from diverse community-based cohorts. This tool is relevant for both African American and non-Hispanic White males and females within the age bracket of 40 to 79 years [18]. It integrates common cardiovascular risk elements including sex, age, SBP, HDL-cholesterol, TC, the presence of hypertension treatment, diabetes status, racial background, and smoking habits into the risk assessment. The calculation of the risk score was performed using a specific calculator available at: https://static.heart.org/riskcalc/app/index.html#!/baseline-risk (accessed on 1 June 2023). No changes were made to the computation procedure.

#### 2.2.4. QRISK3 Risk Calculator (QRISK3)

Introduced in 2017, the QRISK3 calculator serves as a revision of the QRISK2 algorithm, first launched in 2008 and established as the primary risk assessment tool for estimating a 10-year cardiovascular event risk within the English population aged 25 to 84 years [19]. The updated model enhances its predictive capability by incorporating an additional eight risk factors that have been recognized in various studies as potential contributors to CVD. These include the use of corticosteroids, migraine, treatment with atypical antipsychotic drugs, systemic lupus erythematosus, severe mental illness, blood pressure variability, and erectile dysfunction. The calculation was performed using a specific calculator available at: https://qrisk.org/ (accessed on 2 June 2023). No changes were made to the computation procedure.

#### 2.2.5. Framingham Risk Score for Hard Coronary Heart Disease (FRS-hCHD)

The risk score was formulated to predict the risk of coronary heart disease (CHD) events in 10 years, including coronary death and myocardial infarction. It evaluates various risk factors such as sex, age, HDL cholesterol, TC, SBP, smoking status, and hypertension treatment. The FRS-hCHD tool is specifically designed for non-diabetic individuals between the ages of 30 and 79 who have no previous diagnosis of intermittent claudication or coronary heart disease [15]. The calculation was performed using a specific calculator available at: https://www.mdcalc.com/calc/38/framingham-risk-score-hard-coronary-heart-disease (accessed on 1 June 2023). No changes were made to the computation procedure.

#### 2.2.6. Reynolds Risk Score (RRS)

The RRS aims to predict the 10-year likelihood of a major cardiovascular event, including stroke, myocardial infarction, or other significant heart conditions. This score integrates conventional risk determinants like sex, age, cholesterol levels, smoking habits, and blood pressure, along with supplementary biomarkers including high-sensitivity C-reactive protein (CRP) and a history of premature atherosclerosis in the family [20]. While initially formulated for female populations, the model has subsequently been adjusted to apply to male demographics as well. The calculation of the risk score was performed using a specific calculator available at: http://www.reynoldsriskscore.org/ (accessed on 2 June 2023). No changes were made to the computation procedure.

#### 2.2.7. Assessing Cardiovascular Risk Using SIGN (ASSIGN)

The ASSIGN model is tailored to calculate the 10-year likelihood of cardiovascular events in individuals without pre-existing CVD, incorporating measures of social deprivation (via the Scottish Index of Multiple Deprivation) and family history alongside traditional variables [21]. This approach has demonstrated enhanced accuracy compared to other CVD risk scores in forecasting CVD risk within the Scottish population. Moreover, the ASSIGN score is the recommended tool for CVD risk assessment by the Scottish Government Health Directorates and the Scottish Intercollegiate Guidelines Network (SIGN). The calculation of the risk model was performed using a specific calculator available at: https://www.assign-score.com/estimate-the-risk/visitors/ (accessed on 2 June 2023). The Scottish Index of Social Deprivation was not applicable during the computation procedure.

#### 2.2.8. Australian CVD Risk Score (AusCVDRisk)

The AusCVDRisk model was developed to predict the 5-year probability of cardiovascular events, specifically for individuals aged 30 to 79 years without established CVD and who do not fulfill criteria for high risk. This tool is based on the NZ PREDICT-1 equation, originating from an extensive and modern primary care cohort study in New Zealand [22]. The equation has undergone recalibration to suit the Australian demographic and has been adjusted to align with the Australian healthcare framework. The calculation of the risk score was performed using a specific calculator available at: https://www.cvdcheck.org.au/calculator (accessed on 2 August 2023). The inclusion of a postcode variable was not applicable during the computation procedure.

#### 2.2.9. Multi-Ethnic Study of Atherosclerosis (MESA) Risk Score

The MESA risk score is formulated to calculate the 10-year likelihood of cardiovascular events in individuals aged 45 to 85 years who have no diagnosed CVD [23]. This score is derived from the extensive data collected in the MESA study, encompassing over 6800 participants from diverse racial and ethnic backgrounds. The findings from the MESA study confirm the score’s accuracy in assessing CVD risk across a broad spectrum of races and ethnic groups. The calculation of the risk score was performed using a specific calculator available at: https://www.mesa-nhlbi.org/MESACHDRisk/MesaRiskScore/RiskScore.aspx (accessed on 2 June 2023). The coronary artery calcification index was excluded from the computation procedure as this variable was not present in our dataset.

### 2.3. Definitions of Variables

**Figure 1:**
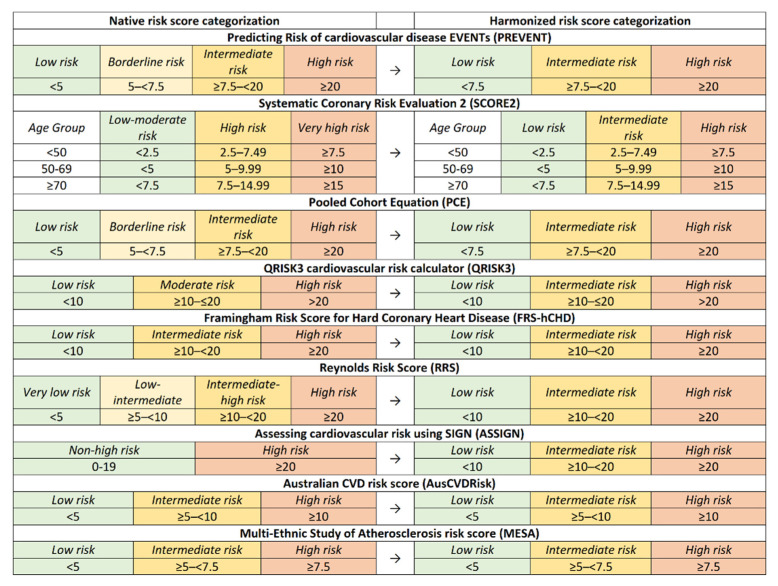
Comparative presentation of native and harmonized cardiovascular risk categorizations after adjustment.

Every Risk Prediction Model (RPM) features distinct risk categories and scoring ranges, requiring the adoption of a standardized methodology to ensure analytical uniformity. Figure 1 graphically depicts these risk categories and their corresponding intervals among the various models. In certain RPMs, the categorization boundaries were maintained, but the terminology was modified for consistency. For example, the ASSIGN risk score initially classified some individuals as “non-high risk”, which was later segmented into low- and intermediate-risk categories to achieve analytical harmony. Similarly, in scores like PREVENT, the term “borderline risk” was redefined as “intermediate risk” to conform to the uniform categorization scheme used in this analysis. This standardization of risk categories enables a more detailed and nuanced comparison.

### 2.4. Statistical Analysis

In the quantitative evaluation, agreement among nine cardiovascular risk prediction models was rigorously examined utilizing a multifaceted methodology. An initial analysis employing descriptive statistics mapped out the frequency distributions of risk categories across each algorithm, establishing a baseline for comprehending the segmentation of patient populations into distinct risk levels. Concordance among model pairs was quantitatively ascertained using Cohen’s Kappa statistics, a robust measure for evaluating categorical agreement, offering a scale from −1 to 1, with higher values denoting greater agreement.

A heatmap was created to enhance the interpretability of the Kappa coefficients. Additional analysis of the model interrelations was facilitated by hierarchical clustering. Utilizing the pairwise Kappa coefficients, a dendrogram was synthesized via the Ward method, aimed at reducing within-cluster variance, thereby categorizing models with similar risk differentiation patterns.

An overarching analysis of score agreement was conducted via Collective Model Agreement Analysis, calculating the frequency of patients categorized consistently across models, thus offering comprehensive insights into agreement levels in a clinical setting.

Statistical analyses were conducted using IBM SPSS software version 25.0 (SPSS, Chicago, IL, USA) or Python, employing libraries such as Pandas for data management, scikit-learn for statistical analysis, and Matplotlib and Seaborn for graphical visualizations. The significance threshold for statistical inferences was established at 0.05.

### 2.5. Ethical Considerations

The study received approval from the Vilnius Regional Biomedical Research Ethics Committee (permission no. 2019/3-1104-603, approval date 26 March 2019).

## 3. Results

### 3.1. Descriptive Statistics

In the current study, within the LitHiR cohort, a total of 6527 participants were assessed, all of whom were female (100%). The mean age of these subjects was 57.62 ± 4.21 years. Noteworthy cardiovascular risk factors were identified; the average body mass index (BMI) was recorded at 31.76 ± 4.68 kg/m^2^, with the TC level averaging 6.33 ± 1.40 mmol/L. The detailed lipid profiles indicated an average LDL cholesterol of 4.13 ± 1.23 mmol/L and an HDL cholesterol of 1.33 ± 0.31 mmol/L. Further, the study population demonstrated a mean SBP of 137.15 ± 15.96 mmHg and a mean DBP of 80.99 ± 10.38 mmHg. The prevalence of comorbidities included 20.3% (*n* = 1325) of the participants diagnosed with diabetes mellitus, 28.5% (*n* = 1861) receiving treatment for hypertension, and 11.7% (*n* = 764) on dyslipidemia management with statins. Additionally, the cohort featured current smokers (*n* = 841, 12.9%) and ex-smokers (*n* = 189, 2.9%), underscoring the multifaceted nature of cardiovascular risk within this cohort. This thorough baseline characterization lays the foundation for subsequent comparative analyses of cardiovascular RPMs (Table 1).

### 3.2. Risk Category Distribution

**Figure 2:**
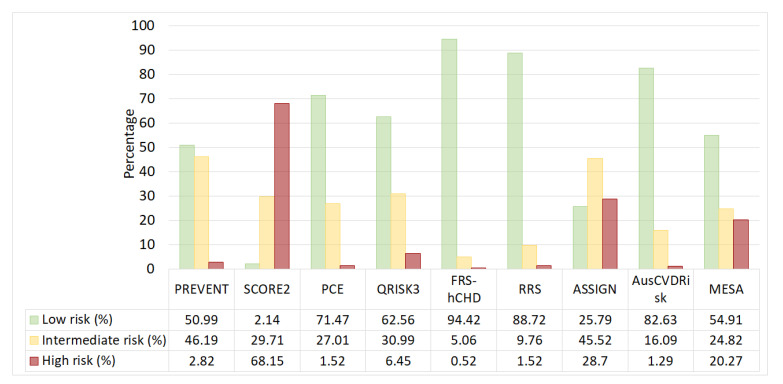
Distribution of cardiovascular risk categories among nine cardiovascular risk prediction models.

The comparative analysis revealed distinct disparities in cardiovascular risk categorization across models (Figure 2). The SCORE2 model had stood out by classifying a majority (*n* = 4448, 68.15%) as high-risk, whereas PCE (*n* = 4665, 71.47%) and AusCVDRisk (*n* = 5393, 82.63%) predominantly identified individuals as low-risk, reflecting a conservative approach in risk stratification. In contrast, ASSIGN displayed a balanced distribution, with a notable presence across all risk categories, suggesting a middle ground in risk assessment. Notably, models such as RRS (*n* = 99, 1.52%) and FRS-hCHD (*n* = 34, 0.52%) allocated a considerably smaller fraction to high-risk compared to SCORE2, MESA (*n* = 1323, 20.27%), and ASSIGN. The PREVENT model, central to the analysis, categorized patients with a discerning balance, with a significant number identified as intermediate (*n* = 3015, 46.19%) and low risk (*n* = 3328, 50.99%), avoiding the extremities observed in SCORE2 and FRS-hCHD.

### 3.3. Pairwise Agreement Analysis

**Figure 3:**
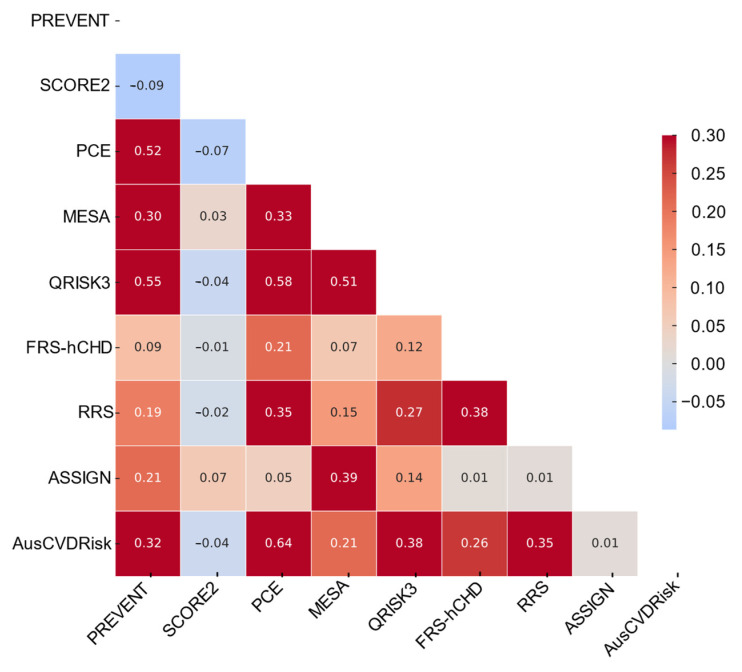
Heatmap illustrating pairwise agreement across nine cardiovascular risk prediction models.

The heatmap of Cohen’s Kappa statistics had revealed the degree of agreement among the cardiovascular risk prediction models (Figure 3). The range of values extends from −1 to 1, with higher values indicative of stronger concordance. A significant observation was the moderate agreement between PREVENT and QRISK3 (κ = 0.55) as well as PREVENT and PCE (κ = 0.52), indicating a similar pattern in patient categorization between these models, suggesting that they may have shared underlying risk assessment methodologies. In contrast, PREVENT demonstrated minimal to no agreement with SCORE2 (κ = −0.09), highlighting a starkly different approach in risk stratification between these models.

Other notable agreements included PCE and AusCVDRisk (κ = 0.64), showcasing a high concordance and potential interchangeability for risk assessment purposes. Additionally, moderate agreements were observed between MESA and ASSIGN (κ = 0.39) and QRISK3 and AusCVDRisk (κ = 0.38), suggesting these pairs of models had similarities in risk categorization but retained distinct methodologies.

### 3.4. Cluster Analysis: Hierarchical Clustering

**Figure 4:**
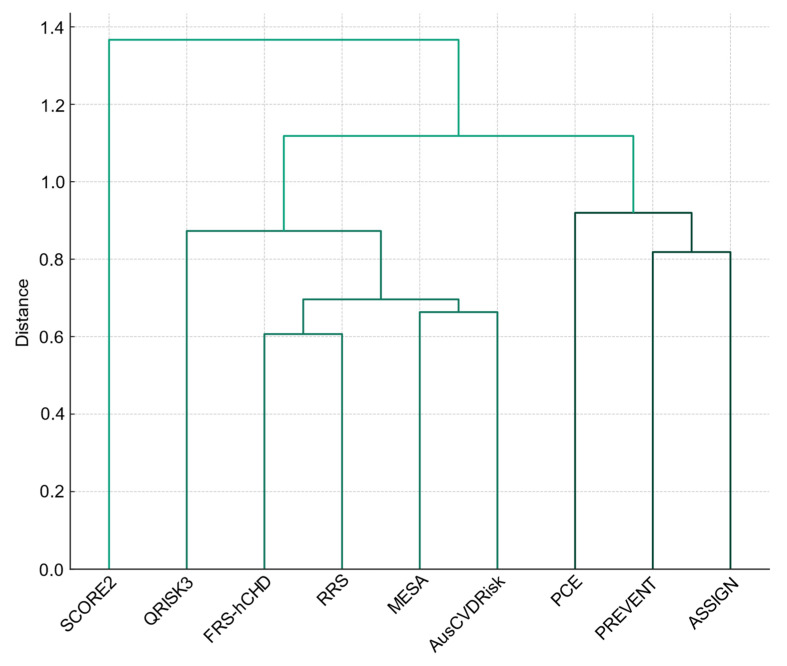
Dendrogram of hierarchical clustering demonstrating similarities across nine cardiovascular risk prediction models.

The hierarchical clustering dendrogram, based on the pairwise Cohen’s Kappa statistics, delineates distinct clusters among the CVD RPMs, illustrating the underlying similarities and differences in their risk stratification approaches. Specifically, three principal clusters can be discerned (Figure 4). The first encompasses the PREVENT model alongside ASSIGN and PCE, underscoring a noteworthy congruence in their approaches to evaluating CVD risk. This clustering suggests that, despite their methodological differences, these models share a common ground in their assessment criteria, pointing towards a nuanced approach in evaluating CVD risk.

A second, broader cluster includes the MESA and AusCVDRisk models, along with RRS and FRS-hCHD as well as QRISK3. Despite the diversity within this group, the models exhibit sufficient commonality in risk categorization, suggesting a generally cohesive yet varied approach to cardiovascular risk prediction across these models.

Distinctively, the SCORE2 model forms an isolated cluster, markedly separate from the others. This segregation accentuates SCORE2’s divergent risk stratification methodology, starkly contrasting with the more congruent approaches observed among the other models, including PREVENT.

### 3.5. Collective Model Agreement Analysis

Finally, we quantified the frequency of agreement among all models in classifying the same patient into a specific risk category, offering an overall perspective on model concordance. Table 2 displays the frequency of occurrences where a designated count of scores concur regarding the risk category for a particular patient. The highest concordance was observed when eight models agree, covering 23.76% of the patient population, followed closely by instances where six models agree, accounting for 20.07% of patients. This analysis highlights the variability in cardiovascular risk assessment across different models, with a substantial portion of cases showing agreement among a majority of the models, yet a complete consensus (all nine models agreeing) is relatively rare, affecting only 1.98% of patients.

## 4. Discussion

This study provides an in-depth comparison of the performance of nine prevalent cardiovascular risk prediction models within a Lithuanian high-risk cohort among women. The analysis reveals a significant variation in how these models classify cardiovascular risk, highlighting the importance of careful application in clinical settings and showcasing the attributes of the newly introduced PREVENT model.

In assessing the categorization of risk, the PREVENT model demonstrated a balanced approach in the context of the various risk stratification methods employed by the analyzed models, offering a unique viewpoint relative to its counterparts. Notably, QRISK3 and MESA presented diverse risk distributions, with QRISK3 identifying a lower risk in a manner akin to PREVENT, while SCORE2 allocated a significant portion (68%) of participants to the high-risk category, and FRS-hCHD predominantly (94%) assigned subjects to the low-risk group, illustrating the broad spectrum of risk assessment practices.

The analysis of model agreement highlights the PREVENT model’s distinctive alignment within the array of cardiovascular risk predictors, showing substantial concordance with models such as QRISK3 (κ = 0.55) and PCE (κ = 0.52), while it shows complete discordance with SCORE2 (κ = –0.09). In addition, the study identifies a notably high degree of agreement between the PCE and AusCVDRisk scores (κ = 0.64), as well as between QRISK3 and PCE (κ = 0.58) and MESA and QRISK3 (κ = 0.51), suggesting these models similarly classify patients. Conversely, SCORE2’s agreement with other models ranged from negative to low (κ from –0.09 to 0.07), pointing to a distinct approach to risk classification. Not to mention, a complete consensus, where all nine models agree is especially rare, affecting only 1.98% of cases. These findings emphasize the diverse tendencies of the models to assign individuals to various risk levels, underlining the critical need for nuanced interpretation of their predictive power within the LitHiR cohort. Moreover, cluster analysis via the dendrogram further highlights the compatibility of the PREVENT model with QRISK3 and PCE, indicating that in certain clinical contexts, these models could be used interchangeably with minimal impact on risk categorization. The clustering of these models suggests that they share a common foundation of traditional cardiovascular risk factors—such as age, cholesterol levels, and blood pressure—while also incorporating novel risk factors like socioeconomic status (in the case of QRISK3) or kidney function (in the case of PREVENT). These additional parameters enhance the model’s ability to capture nuanced risk profiles, particularly in populations with complex socio-economic or metabolic backgrounds, such as the cohort studied here. In contrast, models such as SCORE2 and AusCVDRisk, which form separate clusters, reflect more region-specific approaches to cardiovascular risk assessment. SCORE2 is tailored to European populations, placing a stronger emphasis on high-risk classification and fatal cardiovascular events, whereas AusCVDRisk, calibrated for the Australian population, tends to categorize more individuals as low-risk. This divergence highlights how regional guidelines and baseline population risks significantly influence the performance of these models.

Unsurprisingly, the agreement outcomes align closely with our prior findings from the same study population, which encompassed both male and female participants. Specifically, the subpopulation of women exhibited stronger agreements, with notably higher concordance between MESA and QRISK3. Conversely, there was a reduced level of agreement between the AusCVDRisk and QRISK3 RPMs [24].

Several factors may account for the observed discrepancies in concordance across the CVD RPMs in our investigation. Primarily, the differences in risk classification thresholds each model employs are a prominent contributor. Additionally, variations in model endpoints, arising from their focus on either specific or more general cardiovascular events, different predictive intervals (5 or 10 years), and the unique risk factors each model considers, result in personalized risk assessments for various population segments and clinical contexts across the models [6]. However, the basis of agreement on risk categories rather than absolute risk values may render this point less relevant. Differences in CVD risk categorization may also arise from the varied source cohorts underpinning each risk assessment tool, including those with specific gender focuses, such as the RRS, which was originally developed for female populations [20].

The SCORE2 model, derived from multiple European cohorts [17], and the ASSIGN score, which uses Scottish data and incorporates social deprivation factors [21], exemplify the geographic diversity in cardiovascular risk prediction tools. The AusCVDRisk, based on Australian populations, and the FRS-hCHD, originating from the Framingham Heart Study cohort in the United States [15], further highlight this diversity. Similarly, the MESA tool addresses diverse ethnic groups within the United States [23], while both the PCE and PREVENT models integrate data from multiple cohorts to provide race- and sex-specific risk estimations with the PREVENT model extending its assessment to include factors such as social deprivation, urine albumin–creatinine ratio, and HbA1C, thereby broadening its analytical scope [12,18]. The QRISK3 calculator, developed from a comprehensive UK-based cohort, integrates a broad spectrum of risk factors [19]. Lastly, the US-based RRS incorporates biochemical variables and a family history of premature coronary heart disease with traditional risk factors, reflecting its unique formulation [20].

In the synthesis of findings, it is imperative to address the multifaceted nature of cardiovascular risk assessment and its inherent challenges. The evidence gleaned from the literature underscores a persistent underestimation of cardiovascular risk in women, a demographic traditionally marginalized in cardiovascular research. This underestimation is particularly pronounced in the context of conditions like non-alcoholic fatty liver disease and amid common risk factors such as hypertension and dyslipidemia, which are often overlooked or underestimated in women [25,26,27]. Such findings illuminate the gender-specific disparities in cardiovascular risk assessment and highlight the necessity for refined risk prediction models that accurately reflect the cardiovascular risk profiles of women.

The incorporation of age as a determinant of risk underestimation, particularly in urban women, alongside the analysis of the Framingham score’s inadequacy in accurately assessing risk in women compared to men [28], further accentuates the complexity of cardiovascular risk assessment in women. The evaluation of the SCORE model in the context of postmenopausal women and the examination of the PCE model across diverse populations, including the Women’s Health Initiative, signal an ongoing effort to enhance the precision of risk prediction in women [29,30].

However, the QRISK3 calculator’s superior performance in discriminating ASCVD risk among women, as compared to other assessment tools, underscores the evolving landscape of risk prediction methodologies and their variable applicability across different demographic segments [31]. The findings relating to the underestimation of true aortic SBP by noninvasive blood pressure measurements in women further compound the challenges in achieving accurate cardiovascular risk assessment in this group [32].

While the FRS exhibits tendencies to overestimate risk in populations outside the USA, recalibration efforts have yielded mixed outcomes, with variations in risk estimation accuracy between genders and across ethnic groups [33,34]. The RRS, on the other hand, has demonstrated improved accuracy in classifying intermediate-risk women and has been validated in predicting long-term cardiovascular risk in specific cohorts, including those with inflammatory joint diseases [35,36].

The overarching narrative emerging from a systematic review of various risk prediction algorithms reveals a landscape marked by inconsistency and variability in performance metrics such as discrimination, calibration, and reclassification [37]. This inconsistency is further exemplified by the discordance in risk estimation across different populations, as highlighted by the underestimation and overestimation tendencies of the SCORE, PCE, and other models in various demographic settings [34,38,39]. While the models analyzed in this study offer valuable tools for estimating cardiovascular risk, their predictive capacity is inherently limited by the exclusion of psychosocial determinants such as social support, mental health, and socioeconomic status. These factors are increasingly recognized for their role in shaping cardiovascular outcomes yet remain underrepresented in most current prediction models. The ASSIGN, AusCVDRisk, and PREVENT scores are among the few that incorporate such factors, yet even these models do not capture the full spectrum of influences on health.

The disparity in eligibility criteria for lipid-lowering therapy across distinct risk prediction models, as illustrated by Mortensen et al. [40], encapsulates the broader issue of model agreement and the implications for clinical decision-making. The low level of concordance between different risk models, compounded by methodological variations and demographic specificities, underscores a critical gap in our ability to uniformly assess cardiovascular risk, particularly among women, since in our study, we observed that SCORE2 classified the majority (68.15%) of the cohort as high-risk, while FRS-hCHD (94.42%) and AusCVDRisk (82.63%) predominantly identified individuals as low-risk. This significant variation in risk categorization is critical, as it directly influences treatment decisions, such as the initiation of statin therapy. For example, under SCORE2, a patient classified as high-risk may be recommended for more aggressive preventive measures, such as high-intensity statin therapy or even consideration for additional treatments like ezetimibe or PCSK9 inhibitors. On the other hand, the same patient might be categorized as low-risk by PCE or AusCVDRisk, potentially leading to a recommendation for lifestyle modification alone, without pharmacological intervention. This could result in undertreatment or overtreatment depending on the model used, with profound implications for long-term cardiovascular outcomes. Furthermore, these discrepancies in risk classification could lead to patient confusion or non-compliance if different healthcare providers use different models to assess risk. The variability in categorization underscores the need for clinicians to carefully choose the most appropriate model based on the population being treated and the clinical context. In particular, models such as SCORE2, which are calibrated for high-risk European populations, may prioritize more aggressive prevention strategies, while PCE and AusCVDRisk, which are more conservative in their risk estimates, may be more suitable for populations with lower baseline cardiovascular risk.

Given these insights, it becomes apparent that the journey towards optimizing cardiovascular risk prediction in women necessitates a multifaceted approach. This includes the refinement of existing models to better capture the unique risk profiles of women, the development of gender-specific risk assessment tools, and an overarching commitment to integrating gender-specific considerations into cardiovascular research and clinical practice. Only through such dedicated efforts can we hope to bridge the gap in cardiovascular risk assessment and ensure equitable, precise, and personalized care for women.

### Study Strengths and Limitations

This study distinguishes itself through an extensive comparison of nine distinct cardiovascular risk prediction models, a breadth of analysis that is rare in existing literature. The robustness of this evaluation is further enhanced by the inclusion of a large cohort of 6527 subjects, providing substantial statistical power and bolstering the reliability and applicability of the results. Notably, the study’s cohort comprised exclusively female participants, offering a detailed insight into cardiovascular risk prediction within this demographic. The diversity of the cohort in terms of cardiovascular risk factors—such as hypertension, diabetes, and dyslipidemia—offers a realistic snapshot of the patient demographics typically seen in clinical settings. The analytical rigor of the study is also heightened through the use of advanced statistical methods, including Cohen’s Kappa coefficients to gauge the concordance between models. This study is novel in its inclusion of the latest PREVENT equation model along with other recent RPMs like SCORE2, QRISK3, the updated AusCVDRisk score, and the latest version of PCE, thereby enhancing the timeliness and comprehensiveness of our analysis.

Although the reliance on data from a single large tertiary hospital might be perceived as a limitation due to potential concerns over external validity, it also serves as a strength by ensuring consistency in data collection methods and reducing variations across facilities that might lead to confounding factors. Nonetheless, the single-center approach constitutes a primary limitation, potentially affecting the representativeness of the findings for a wider population. The exclusive focus on a female cohort introduces another limitation, as it may not fully capture the gender-specific nuances in cardiovascular risk prediction, underscoring the need for future studies to include both male and female participants to enhance the generalizability of the findings. Furthermore, the study examines a cohort exclusively composed of individuals diagnosed with metabolic syndrome. This specific focus may restrict the broader applicability of the results, highlighting the necessity for future research to encompass a more varied participant pool. The exclusive selection of participants with metabolic syndrome narrows the study’s relevance, suggesting that these findings should be extrapolated with caution to a more diverse and general population. Additionally, the cross-sectional design of the study limits the ability to determine causality or track changes in risk assessments over time. The lack of data on actual cardiovascular events constrains our capacity to validate the predictive accuracy of the scores examined. Although the absence of longitudinal follow-up is a noted shortcoming, this aspect is slated for exploration in subsequent studies aimed at evaluating the predictive performance of the models discussed. While the large cohort size and the array of cardiovascular risk profiles examined are strengths, the possibility of unaccounted confounding factors remains. Furthermore, the tendency of different models to categorize individuals into specific risk tiers necessitates careful consideration when interpreting and applying these models interchangeably.

## 5. Conclusions

The selection of a RPM plays a pivotal role in influencing clinical decisions and managing patient care. In the comparison of cardiovascular risk categorization methods, the PREVENT model emerged as a balanced option, steering clear of the extremes seen in both SCORE2 and FRS-hCHD. Notably, the highest level of concordance was observed between the PREVENT model and both the PCE and QRISK3 RPMs. Conversely, the SCORE2 model demonstrated consistently low or negative agreement with other scores, highlighting its unique approach to risk estimation. Remarkably, agreement across all nine models on the same risk category for a patient was rare, occurring in only 1.98% of cases. These findings accentuate the critical need for additional research to assess the predictive accuracy of these models specifically among the Lithuanian female population.

## Figures and Tables

**Table 1:** Baseline characteristics of the study cohort (*n* = 6527).

Characteristics	
Gender: *n* (%)	Female 6527 (100)
Age, years: mean (SD)	57.62 (4.21)
Body mass index, kg/m^2^: mean (SD)	31.76 (4.68)
Systolic blood pressure, mmHg: mean (SD)	137.15 (15.96)
Diastolic blood pressure, mmHg: mean (SD)	80.99 (10.38)
Total cholesterol, mmol/L: mean (SD)	6.33 (1.40)
Triglycerides, mmol/L: mean (SD)	1.88 (1.15)
Low-density lipoprotein cholesterol, mmol/L: mean (SD)	4.13 (1.23)
High-density lipoprotein cholesterol, mmol/L: mean (SD)	1.33 (0.31)
Estimated glomerular filtration rate, mL/min/1.73 m^2^: mean (SD)	88.97 (10.74)
Urine Albumin–Creatinine Ratio, mg/g: mean (SD)	12.79 (100.21)
C-reactive protein, mg/L: mean (SD)	3.09 (3.98)
Creatinine, µmol/L: mean (SD)	65.60 (8.93)
Fasting glucose, mmol/L: mean (SD)	6.30 (1.51)
Diabetes mellitus: *n* (%)	1325 (20.3%)
Dyslipidemia treatment (statins): *n* (%)	764 (11.7%)
Hypertension treatment: *n* (%)	1861 (28.5%)
Antiplatelet treatment: *n* (%)	19 (0.29%)
Current smoker: *n* (%)	841 (12.9)
Ex-smoker: *n* (%)	189 (2.9)

SD—standard deviation.

**Table 2:** The frequency and percentage of occurrences where a designated count of models agree regarding the risk category for a particular patient.

Number of Models Agreeing	Number of Patients	Percentage of Patients (%)
3	177	2.71
4	1214	18.6
5	1039	15.92
6	1310	20.07
7	1107	16.96
8	1551	23.76
9	129	1.98

## Data Availability

Due to the conditions of the bioethical approval, the data supporting the findings of this article are not publicly available, as the approval restricts free data distribution and limits access to specified individuals within the framework of the approval. Data will be made available by the corresponding author upon reasonable request.
